# P-705. Time to Testing, Diagnosis, and Hospitalization Among Pediatric and Adult Patients with RSV in Two Seasons: A Real-World Data Analysis using Electronic Health Records in the US

**DOI:** 10.1093/ofid/ofaf695.917

**Published:** 2026-01-11

**Authors:** Wing Yu Tang, Edward Weinstein, Negar Niki Alami, Lulu Lee, Stacey Purinton, Vicky W Li, Robert J Taylor, Neelanzana Paudel, Sima S Toussi

**Affiliations:** Pfizer Inc., New York, NY; Pfizer Inc., New York, NY; Pfizer Inc., New York, NY; Oracle Life Sciences, Austin, Texas; Oracle Life Sciences, Austin, Texas; Oracle Life Sciences, Austin, Texas; Oracle Life Sciences, Austin, Texas; Oracle Life Sciences, Austin, Texas; Pfizer Inc., New York, NY

## Abstract

**Background:**

Respiratory syncytial virus (RSV) infection is a common respiratory illness, that can lead to severe illness requiring hospitalization. This study aims to understand the time course between presentation of first RSV signs and symptoms to RSV earliest testing, diagnosis, and hospitalization.

**Figure:**
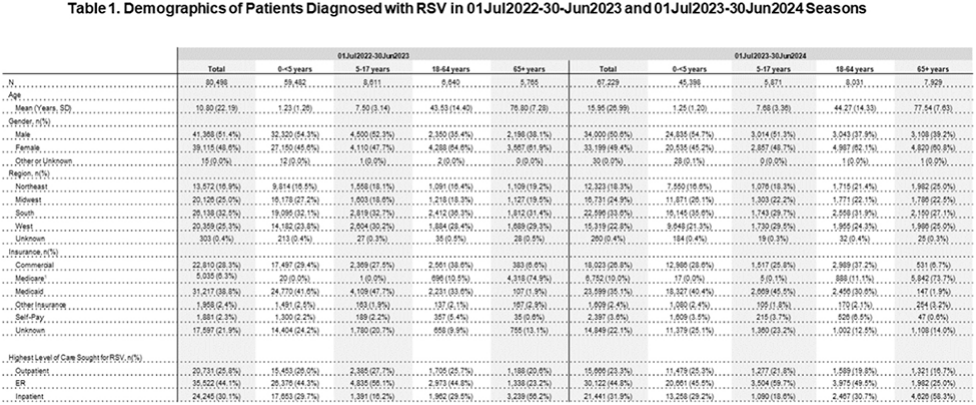

**Figure:**
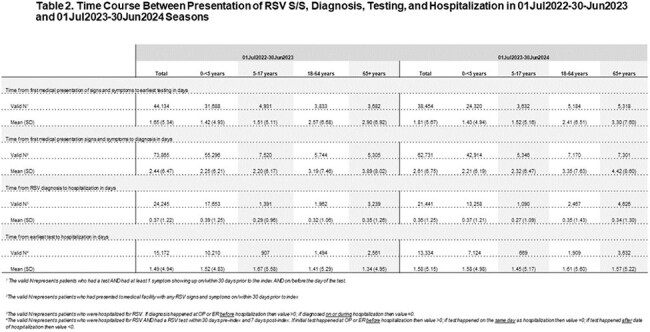

**Methods:**

A retrospective analysis was conducted using structured data from Oracle Life Sciences’ nationally representative electronic health records (EHR) database (∼24 million patients annually; 115 million total). Patients were identified via RSV ICD-9, ICD-10, and/or SNOMED-CT codes within the two seasons of interest (Season 1[S1]: 2022Jul01-2023Jun30 and Season 2[S2]: 2023Jul01-2024Jun30; qualifying once per season). Setting of care was categorized via the highest level of care a patient needed within the season (Inpatient >ER > Outpatient). Descriptive statistics were conducted based on data applicability to examine time to testing, diagnosis, and hospitalization among age groups (0< 5, 5-17, 18-64, 65+YO).

**Results:**

A total of 80,498 RSV patients were identified in S1 and 67,229 for S2 (Table 1). The majority of patients with RSV were 0-< 5 years old (S1 73.9%; S2 67.5%). The inpatient setting had the highest incidence for older adults (65+: S1 56.2%; S2: 58.3%); while ER for younger patients (0-< 5: S1 44.3%; S2: 45.5%). The S1 mean time from presenting for medical care with RSV signs and symptoms (S/S) to testing was 1.65 days(d) overall, with 0-< 5 at 1.42d and 65+ at 2.90d (Table 2). S1 mean time from this RSV S/S to diagnosis was 2.44d overall, with 0-< 5 at 2.25d and 65+ 3.89d. S1 mean time from RSV diagnosis to hospitalization was 0.37d (age strata in Table 2). All findings were similar for S2.

**Conclusion:**

This EHR analysis of patients diagnosed with RSV showed that the diagnosis of RSV occurs 2 to 4 days from the initial time of presenting for medical care with RSV S/S. Those requiring hospitalization were admitted in less than 1 day post diagnosis.

**Disclosures:**

Wing Yu Tang, MPH, Pfizer: Employee|Pfizer: Stocks/Bonds (Public Company) Edward Weinstein, MD, PhD, Pfizer: Employee|Pfizer: Stocks/Bonds (Public Company) Negar Niki Alami, MD, Pfizer: Employee|Pfizer: Stocks/Bonds (Public Company) Lulu Lee, PhD, Oracle Life Sciences: Employed by Oracle Life Sciences, which received funding from Pfizer to conduct the study. Stacey Purinton, MSN, MPH, Oracle Life Sciences: Employed by Oracle Life Sciences, which received funding from Pfizer to conduct the study. Vicky W. Li, MPH, Oracle Life Sciences: Employed by Oracle Life Sciences, which received funding from Pfizer to conduct the study. Robert J. Taylor, AS, Oracle Life Sciences: Employed by Oracle Life Sciences, which received funding from Pfizer to conduct the study. Neelanzana Paudel, MS, Oracle Life Sciences: Employed by Oracle Life Sciences, which received funding from Pfizer to conduct the study. Sima S. Toussi, MD, Pfizer: Stocks/Bonds (Private Company)

